# Cross-generational ripples: sublethal fipronil exposure alters *Binodoxys communis* microbiome without lethal consequences

**DOI:** 10.3389/fmicb.2025.1637234

**Published:** 2025-11-21

**Authors:** Lisha Wang, Weijiao Liu, Li Wang, Kaixin Zhang, Dongyang Li, Jichao Ji, Junyu Luo, Xiangzhen Zhu, Jinjie Cui, Xueke Gao

**Affiliations:** 1 State Key Laboratory of Cotton Bio-breeding and Integrated Utilization, Institute of Cotton Research, Chinese Academy of Agricultural Sciences, Anyang, Henan, China; 2 Zhengzhou Research Base, State Key Laboratory of Cotton Bio-breeding and Integrated Utilization, School of Agricultural Sciences, Zhengzhou University, Zhengzhou, Henan, China

**Keywords:** *Binodoxys communis*, fipronil, sublethal concentration, 16S rRNA, symbiotic bacteria

## Abstract

**Introduction:**

Fipronil, a broad-spectrum phenylpyrazole insecticide, demonstrates high efficacy against *Aphis gossypii* (cotton aphid). However, its potential effects on *Binodoxys communis*, a key natural enemy of A. gossypii, remain largely unexplored. This study comprehensively assessed the safety of fipronil for *B. communis*, with particular emphasis on sublethal effects and associated microbiome alterations.

**Methods:**

We evaluated the sublethal effects of fipronil on the development of *B. communis* across parental (F0) and offspring (F1) generations. Furthermore, the alterations in the microbial diversity and community structure of *B. communis* were analyzed using 16S rRNA sequencing. Functional prediction of the microbiota was performed via PICRUSt2.

**Results:**

Indirect fipronil exposure significantly prolonged larval development in the parental generation (F0, *p* = 0.017), while showing no statistically significant impact on the offspring generation (F1). 16S rRNA sequencing revealed apparent alterations in the microbial community. In adults, the dominant genus shifted from *Akkermansia* to *Muribaculum* after 1 h exposure, while the dominant phylum showed significantly reduced abundance after 3 d. In larvae, the major phylum (*Proteobacteria*) remained unchanged, but the major genus shifted from *Brevitalea* to *Vicinamibacter*. Functional prediction indicated that the predicted genes were predominantly enriched in metabolic pathways (75% of the functional repertoire).

**Discussion:**

These results suggest that fipronil exposure induces previously unrecognized sublethal effects on a key natural enemy insect, primarily by disrupting its symbiotic microbiota, which may play a major role in host metabolism. Our findings highlight the ecological risks of fipronil and emphasize the need for pesticide risk assessments that consider sublethal effects on beneficial insects and their microbiota.

## 1 Introduction

Farmers and pest management programs in certain regions, such as parts of the Americas and Asia, have historically incorporated fipronil into their strategies due to its efficacy against a broad spectrum of agricultural pests (Ascenzi et al., 2018; Guima et al., 2022), particularly demonstrating high toxicity against *Aphis gossypii* Glover (Hainzl and Casida, 1996). Among biological control agents, the parasitoid wasp *Binodoxys communis* Gahan (Hymenoptera: Braconidae) has proven particularly effective against aphid species including *A. gossypii* and soybean aphids (Wyckhuys et al., 2008; Ghising et al., 2012; Yang et al., 2017; Zhang et al., 2020). However, the widespread application of fipronil for aphid control inevitably leads to the exposure of this key parasitoid wasp in the field. This natural enemy exerts control through parasitic behavior that ultimately leads to host mummification, yet whilst some research has begun to examine the sublethal effects of fipronil on *B. communis* (such as developmental suppression), its impact on the parasitoid’s key symbiotic microbiome remains unclear.

The systemic neurotoxicity of fipronil adversely affects beneficial insects and non-target organisms across multiple ecosystems (Pino-Otín et al., 2021; Wazir and Shad, 2022; Sotero et al., 2024). Soil applications have been shown to significantly reduce populations of non-target arthropods (Pisa et al., 2015), while aquatic organisms experience lethal and sublethal effects, such as reduced survival, inhibited growth, and behavioral abnormalities, from environmental contamination (Tingle et al., 2003; Gibbons et al., 2015; Dourado et al., 2023). Studies have demonstrated that exposure to fipronil at doses as low as 0.1 ng/bee (or the 24 h LC₅₀) can induce adverse effects in honeybees, including impaired individual development, aberrant behavioral changes, and disruptions to gut microbiota homeostasis (El Hassani et al., 2005; Farder-Gomes et al., 2021). Furthermore, such sublethal effects have been documented in a broad range of non-target organisms, from essential pollinators and farmland butterflies to laboratory model insects such as fruit flies (Teixeira et al., 2009). These studies collectively demonstrate that the ecological risks associated with fipronil are widespread, and its sublethal effects on non-target insects represent a significant dimension that cannot be overlooked in risk assessments.

Microbial communities, which play pivotal roles in insect physiology and ecosystem functioning (Zhang et al., 2021; Hu et al., 2024; Kelleher and Ramalho, 2025). Fipronil exposure has been documented to alter microbial composition in both soil ecosystems and beneficial insects, with studies demonstrating transient shifts in bacterial, fungal, and ammonia-oxidizing microorganism communities following field applications (Guima et al., 2022; Sim et al., 2023). While recent evidence indicates that sublethal doses of fipronil negatively affect *B. communis* development by altering metabolic pathways leading to reduced parasitism and survival rates (Du et al., 2024), the effects of such insecticides on parasitoid wasp microbiomes remain largely unexplored.

This study evaluated the transgenerational developmental effects of direct and indirect sublethal fipronil exposure (LC_10_, LC_25_) on *B. communis*, assessing larval duration, pupal duration, and total survival time. In addition, 16S rRNA sequencing revealed shifts in the *B. communis* microbial community structure at 1 h and 3 d post-exposure compared to the control. Our findings provide critical insights for developing sustainable integrated pest management strategies that balance chemical control with natural enemy conservation. Furthermore, this work provides a scientific basis for the safe application of pesticides in farmland.

## 2 Materials and methods

### 2.1 Plant and insect materials

The cotton variety CCRI 49 was obtained from the Institute of Cotton Research, Chinese Academy of Agricultural Sciences (CAAS). All experiments were conducted under controlled environmental conditions (26 ± 1 °C, 70 ± 5% RH, 14 L, 10D photoperiod).

The *Aphis gossypii* population used in this study was maintained as a laboratory colony under identical environmental conditions (26 ± 1 °C, 70 ± 5% RH, 14 L, 10D photoperiod). *Binodoxys communis* was originally collected from cotton fields at the CAAS experimental station (36^°^5′34.8”N, 114°31′47.19″E) and subsequently reared in the laboratory. The parasitoid colony was maintained by exposing adults to second-instar *A. gossypii* nymphs under controlled conditions (26 ± 1 °C, 75 ± 5% RH, 14 L, 10D photoperiod). For experiments, we used newly emerged adult wasps (<24 h post-emergence) to ensure age uniformity.

None of the aforementioned experimental materials had been exposed to the pesticide fipronil.

### 2.2 Chemical reagent

The insecticide fipronil (purity ≥98.8%) was provided by Shanghai LGC Science Ltd. (Shanghai, China). All experimental solutions were prepared from this technical grade material.

### 2.3 Determination of fipronil toxicity to *Binodoxys communis* and preparation of *B. communis* specimens

The contact toxicity of fipronil to adult *B. communis* was determined via a residual film bioassay (Desneux et al., 2004). Graded concentrations of fipronil (0, 1, 5, 10, 50, and 100 mg/L) were used to coat the inner surface of finger-shaped tubes (32.73 cm^2^ internal area). After drying under controlled conditions (25 ± 1 °C, 45 ± 5% RH), twenty newly emerged wasps were transferred into each tube. Each treatment was replicated three times, and mortality was scored after 24 h. Assays with control mortality exceeding 10% were discarded.

In this experiment, we collected parasitoid wasps from two exposure routes: (1) Host-mediated exposure: Second-instar cotton aphids were placed on fresh cotton leaves and treated with a sublethal concentration of fipronil or 0.1% Triton X-100 (control) for 1 h. Subsequently, *B. communis* that had emerged within 24 h were introduced for parasitization. Larvae were collected 3 d post-parasitization (after removing adult aphids under a microscope). (2) Direct adult exposure: Newly emerged adult wasps (within 24 h post-eclosion) were exposed to treated residue vials for 1 h, then transferred to clean tubes and provided with 10% honey water as a food source. The treatment group was exposed to a sublethal concentration of fipronil, while the control group was exposed to 0.1% Triton X-100. Adult *B. communis* were collected at 1 h and 3 d post-treatment. For each treatment group, thirty surviving individuals (constituting biological replicates) were transferred to sterile, enzyme-free centrifuge tubes. Samples were immediately flash-frozen in liquid nitrogen to preserve microbial DNA integrity and subsequently stored at −80 °C until further processing. From the assembled samples, three independent biological replicates per treatment were randomly selected for 16S rRNA gene sequencing analysis.

### 2.4 Effects of fipronil on the growth and development of *B. communis*

#### 2.4.1 Effects of sublethal concentrations of fipronil on cotton aphids on parasitoid larvae (indirect exposure to fipronil)

Second-instar nymphs of the cotton aphid (*A. gossypii*) were exposed to sublethal concentrations of fipronil (LC₁₀: 1.19 mg/L and LC₂₅: 1.73 mg/L; Du et al., 2024) for 1 h, with a control group treated using a fipronil-free diluent solution (0.1% Triton X-100). Following exposure, aphids were transferred to agar plates (containing 1.8% agar) with clean cotton leaves for rearing. Newly emerged female *B. communis* adults were then introduced and allowed to parasitise for 8 h before removal. Parameters including larval duration, pupal duration, and total development period of the F0 generation parasitoid were recorded. Subsequently, F0 generation adults emerging within 24 h were collected from each treatment group. Their progeny (F1 generation, untreated with fipronil) underwent identical assessment of the aforementioned parameters. Each treatment comprised 30 aphids, with three replicates.

#### 2.4.2 Effects of sublethal concentrations of fipronil on adult *B. communis* (direct exposure to fipronil)

Thirty newly emerged *B. communis* adults were placed in fipronil-impregnated film tubes at sublethal concentrations (LC_10_ and LC_25_) for 1 h, with an unexposed group serving as control. Following treatment, the parasitoids were transferred to fresh leaves (containing 1.8% agar) in Petri dishes housing 30 s-instar aphid nymphs. Leaves were replaced every three days to maintain normal aphid growth. The larval period, pupal period, and total survival time of the F0 generation parasitoid were recorded. Adults mated within 24 h post-eclosion were collected, and identical measurements were performed on their F1 generation (untreated). Each treatment group was replicated three times.

### 2.5 DNA extraction and PCR amplification

Total genomic DNA was extracted from surface-sterilized insect samples (sequentially treated with 75% ethanol for 30 s and 3% hydrogen peroxide for 45 s (Du et al., 2024), followed by three sterile water rinses) using the TIANamp Genomic DNA Kit (TIANGEN, China). DNA concentration and purity were quantified with a NanoDrop 2000C (Thermo Scientific, USA), and integrity was confirmed via 1.5% agarose gel electrophoresis. To monitor exogenous contamination, extraction blanks (reagents without sample) and PCR negatives (nuclease-free water instead of template) were included in each batch. PCR reactions (20 μL total volume) were performed in triplicate to amplify the V3–V4 region of the 16S rRNA gene using primers 338F/806R (5 μM, HPLC-purified). Each reaction contained: 10 ng DNA template, 0.8 μL each of forward and reverse primers, 2 μL dNTPs (2.5 mM), 4 μL 5 × FastPfu buffer, 0.4 μL FastPfu polymerase, and nuclease-free water. The thermal cycling conditions were as follows: 95 °C for 3 min; 27 cycles of 95 °C for 30 s, 55 °C for 30 s, and 72 °C for 45 s; final extension at 72 °C for 10 min. The resulting amplicons were visualized electrophoretically, purified, and normalized for sequencing.

### 2.6 Data analysis

The original 16S rRNA sequencing data were processed using QIIME 2 (v2020.2). To maintain comparability with conventional OTU-based studies, sequences were clustered into operational taxonomic units (OTUs) at a 97% similarity threshold using UPARSE (v7.0.1001). Representative sequences from each OTU were then taxonomically annotated using the SILVA database. Alpha diversity was assessed using the Chao1 index (richness) and the Shannon index (diversity). Beta diversity was evaluated by visualizing principal component analysis (PCA) plots using the R package ade4, while Venn diagrams were employed to illustrate OTUs shared between groups and those unique to each group. The functional potential of the microbial communities was predicted from the 16S rRNA gene sequences using PICRUSt2. For the alpha diversity indices and biometric data (including larval duration, pupal duration, and total survival time), a one-way analysis of variance (ANOVA) followed by post-hoc tests (LSD test or Games-Howell test, as appropriate) was applied if the data met the assumptions of parametric tests, which were verified using the Shapiro-Wilk test for normality and either Bartlett’s or Levene’s test for homogeneity of variances; otherwise, the Kruskal-Wallis H test was used. All statistical analyses were performed using SPSS Statistics (version 27.0). Graphical representations of the data were generated with GraphPad Prism (version 9.0.0).

All data analyses were conducted using SPSS 27.0. Probability regression analysis was employed to calculate the LC_10_ and LC_25_ values for sublethal and intergenerational toxic effects. Differences in larval stage survival, pupal stage survival, and total survival time between treatment groups were compared using one-way analysis of variance (ANOVA). Prior to analysis, data normality (Shapiro–Wilk test) and homogeneity of variance (Levene’s test) were verified. Where data met normality and homogeneity of variance criteria, significant ANOVA results (*p* < 0.05) underwent post-hoc LSD comparisons; where data were normally distributed but heterogeneous in variance, Games-Howell tests were employed for post-hoc analysis. Where data failed to satisfy the normality assumption, the non-parametric Kruskal-Wallis H test was employed. The significance level for all tests was set at *p* < 0.05.

## 3 Results

### 3.1 Determination of fipronil toxicity to *B. communis*

The contact toxicity of fipronil to *B. communis* was evaluated using a residual film bioassay. This assay determined the LC10 and LC25 values to be 0.34 mg/L (95% CI: 0.16 – 0.54 mg/L) and 0.64 mg/L (95% CI: 0.37 – 0.91 mg/L), respectively (Table S2). These two sublethal concentrations (LC₁₀ and LC25) were subsequently selected for evaluating the non-lethal effects of fipronil on *B. communis*.

### 3.2 Effects of fipronil on *B. communis* across generations

**Figure 1:**
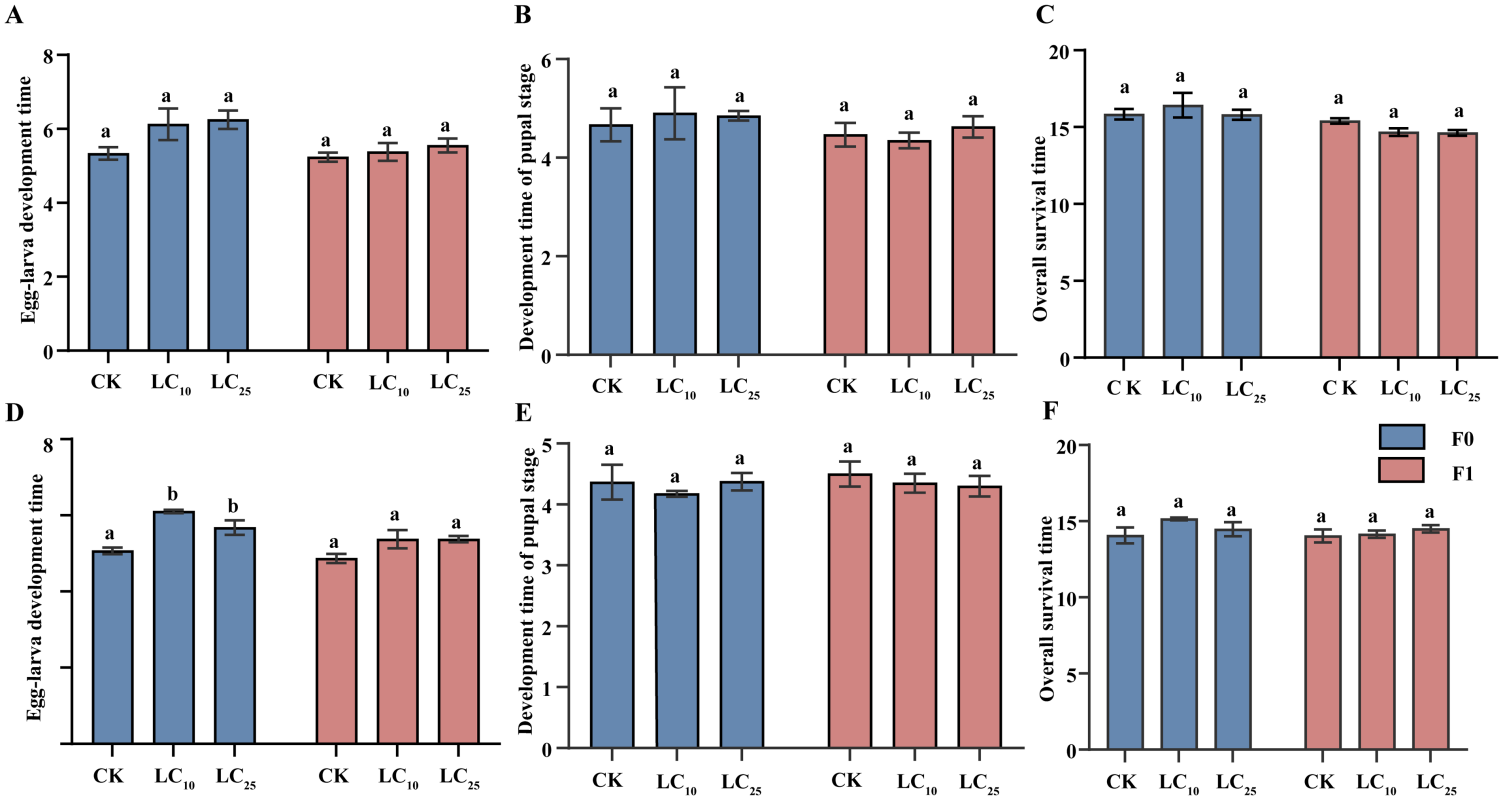
Effect of sublethal concentrations of fipronil on two consecutive generations of *B. communis*. F0, parental generation; F1, offspring of exposed insects. **(A-C)** Effects of direct exposure to fipronil on *B. communis.* **(D-F)** Effects of indirect exposure to fipronil on *B. communis*. Data are presented as the Mean ± SE, LSD test, with different lowercase letters denoting significant differences between treatments (*p* < 0.05).

Direct exposure of *B. communis* to sublethal fipronil concentrations (LC_10_ and LC_25_) showed no significant effects on developmental durations in either generation (Figures 1A–C). The control group (F0 generation) exhibited mean developmental times of 5.33 d (larval), 4.67 d (pupal), and 15.83 d (total). No significant differences were observed in any developmental duration between the control and treatment groups (larval: *p* = 0.285; pupal: *p* = 0.207; total: *p* = 0.212). Similar non-significant patterns were observed in the F1 generation (larval: 5.23 d; pupal: 4.67 d; total: 15.40 d).

However, Sublethal fipronil exposure caused significant developmental delays in the F0 generation, specifically a prolongation of the larval stage (Figures 1D–F). Larval duration increased significantly to 6.10 d (LC_10_) and 5.68 d (LC_25_) compared to controls (5.07 d; *p* = 0.017). While pupal duration showed a non-significant reduction (LC_10_: 4.18 d vs. control: 4.37 d; *F* = 0.516, df = 2.8, *p* = 0.615), total developmental time decreased. The F1 generation displayed concentration-dependent trends in all developmental parameters, though these did not reach statistical significance.

### 3.3 Microbiome profiling of *B. communis*

High-throughput sequencing of the 16S rRNA V3-V4 region generated approximately 3.76 million paired-end reads from 27 *B. communis* samples following quality filtering and chimera removal. After read merging and quality control, 2.16 million high-quality clean reads were obtained (79,200–79,700 reads per sample; mean ± SD: 79,000 ± 1,900; see Table S1 for full metrics).

Rarefaction analysis showed that curves reached plateau phases at approximately 10,000 sequences per sample (Figure S1), suggesting that the sequencing depth was adequate to capture a comprehensive representation of the microbial diversity.

### 3.4 Impact of sublethal fipronil concentrations on *B. communis* larval microbiome

**Figure 2:**
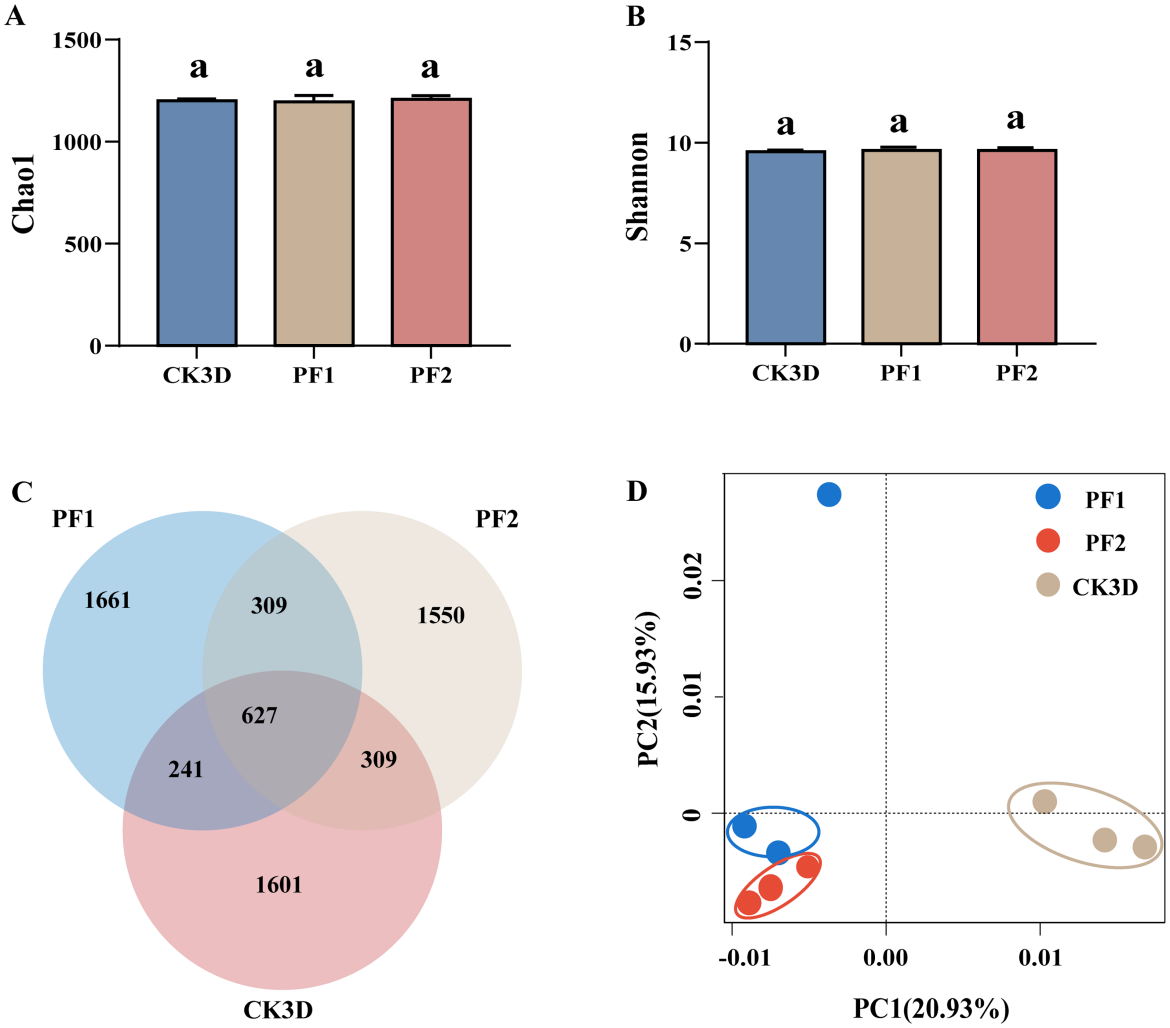
*B. communis* larval symbiotic bacterial community dynamics. **(A,B)** Boxplot of *α*-diversity measured by the second indexes. Letters indicate differences based on LSD test following one-way ANOVA. Different letters indicate significant differences (*P* < 0.05). **(C)** OTU Venn diagram analysis in different samples. **(D)** Principal component analysis (PCA) between different subgroups of samples.

Principal component analysis revealed clear separation between treatment and control groups, indicating concentration-dependent microbiome shifts (Figure 2D). Analysis of alpha diversity showed specific shifts: although species richness (Chao1 index) decreased, community diversity (Shannon index) increased following exposure (Figures 2A,B). Venn analysis identified 627 operational taxonomic units (OTUs) common to all groups, suggesting a stable core microbiome, while the number of unique OTUs varied considerably among treatments (LC_10_: 1,661; LC_25_: 1,150; control: 1,601), indicating selective effects of fipronil (Figure 2C).

**Figure 3:**
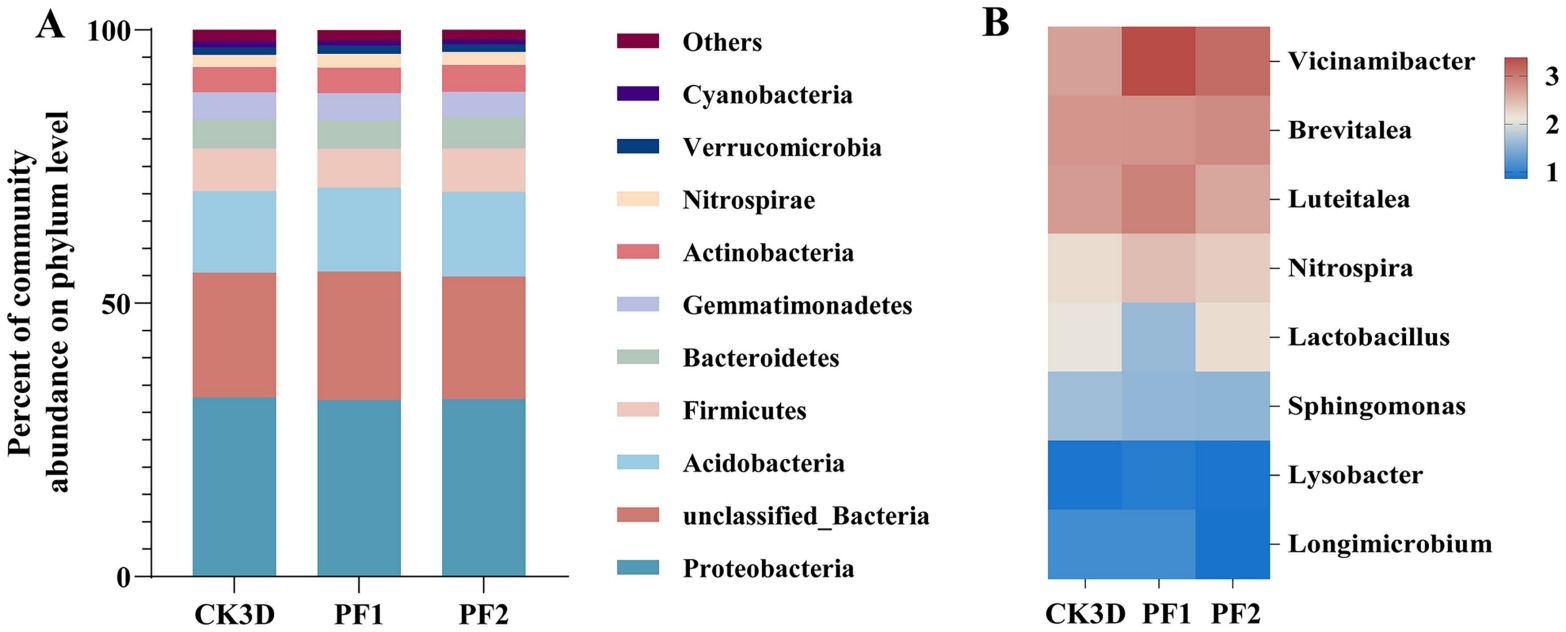
Bacterial composition of larval *B. communis*. **(A)** Relative abundance of the bacterial community at the phylum level. **(B)** Heat map analysis of the bacterial community at the genus level.

Proteobacteria maintained dominance across all treatments (control: 32.79%; LC_10_: 32.27%; LC_25_: 32.47%), followed by sequences that could not be classified at the phylum level (control: 22.82%; LC_10_: 23.52%; LC_25_: 22.41%) and Acidobacteria (Figure 3A). At the genus level, taxonomic profiles normalized by sequencing depth revealed notable shifts in dominant taxa following fipronil exposure. *Brevitalea* was the most abundant genus in the larvae (CK3D: 2.77%), and the dominant genus changed after fipronil treatment *(Vicinamibacter)* (LC_10_ 3.37%, LC_25_ 3.11%). The relative abundance of *Vicinamibacter* was significantly higher than in the control group (2.69%, *p* = 0.006, *p* = 0.004). At sublethal concentrations (LC_10_, LC_25_), the relative abundances of *Vicinamibacter*, *Brevitalea*, and *Nitrospira* were all higher than in the control group (2.69, 2.77, 2.73%). The relative abundance of *Sphingomonas* was lower than in the control group (1.65%). Specifically: *Vicinamibacter* and *Nitrospira* exhibited higher relative abundances at LC_10_ (3.37, 2.46%) than at LC_25_ (3.11, 2.34%). It was also found that the relative abundance of the dominant genus *Brevitalea* increased with increasing sublethal concentration, in contrast to *Vicinamibacter* (Figure 3B). These results demonstrate that sublethal fipronil exposure induces both quantitative and qualitative changes in the larval microbiome of *B. communis*.

### 3.5 Impact of fipronil on the bacterial community in adult *B. communis*

**Figure 4:**
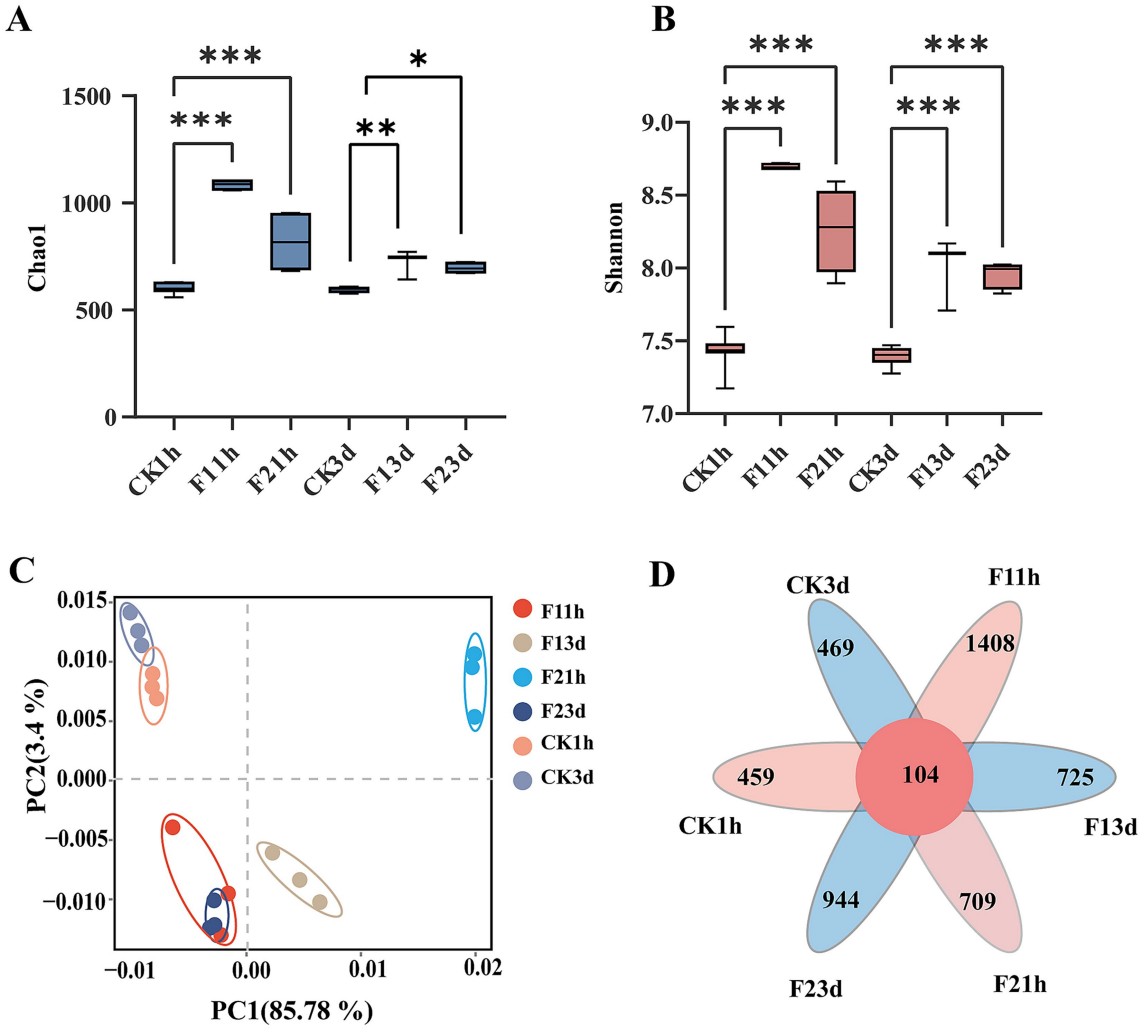
Bacterial community dynamics in adult *B. communis* symbionts. **(A,B)** Box plots of alpha diversity measured by second indices (Letters indicate differences based on LSD test following ANOVA. Data are presented as mean ± SE, *P < 0.05, **P < 0.01, ***P < 0.001). **(C)** Principal component analysis (PCA) between samples from different subgroups. **(D)** OTU Venn diagram analysis of different samples.

Microbial richness (Chao1 indice) significantly increased in adult *B. communis* following fipronil exposure (1 h and 3 d) compared to controls (*p* < 0.05), with LC10 treatments showing greater effects than LC25. Diversity indices (Shannon) were also significantly elevated in exposed wasps (*p* < 0.05, Figures 4A,B). Principal component analysis revealed distinct separation between fipronil-treated and control groups at both time. The first two principal components (PC1 and PC2) together explained 89.19% of the total variance in the microbial community data (PC1: 85.79%; PC2: 3.4%), supporting that the observed separation is a major source of variation in the dataset (Figure 4C). OTU analysis revealed distinct, treatment-specific microbial community patterns. The number of unique OTUs within each treatment group varied significantly: at the 1 h exposure time point, counts were 459 (control), 1,408 (F1), and 944 (F2); this shifted to 469 (control), 725 (F1), and 709 (F2) after 3 days of exposure. A shared microbiome comprising of 104 OTUs was found to be persistently present across all treatment groups and time points (Figure 4D), indicating a stable microbial component resistant to the applied treatments.

**Figure 5:**
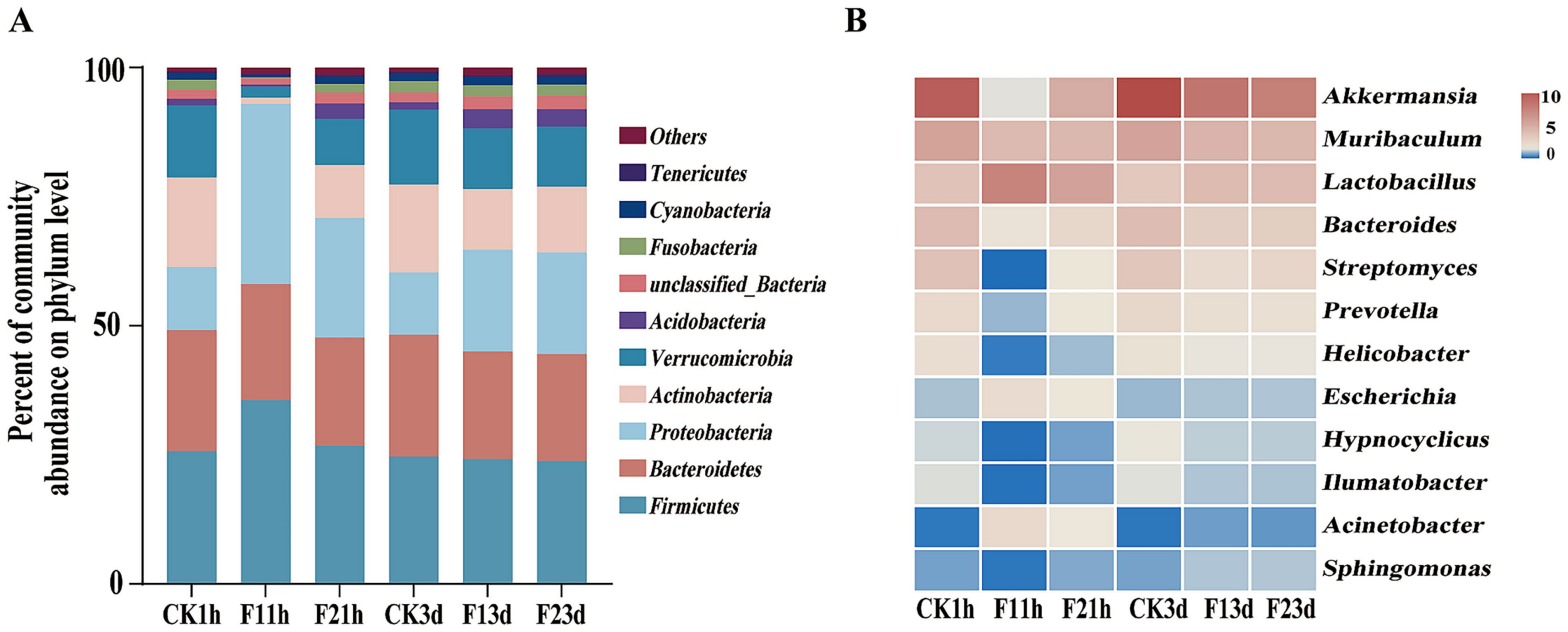
Analysis of the bacterial composition of adult *B. communis*. **(A)** Relative abundance of bacterial communities at the genus level. **(B)** Heat map analysis of bacterial communities at the genus level.

Five phyla dominated the adult microbiome (*Firmicutes, Bacteroidetes, Proteobacteria, Actinobacteriota,* and *Verrucomicrobia*), collectively representing >85% of bacterial communities. Following fipronil exposure, *Firmicutes* remained the dominant phylum, with its relative abundance increasing from 26.15% (CK1h) to 35.62% (LC_10_) and 28.74% (LC_25_) after 1 h (*p* = 0.013, *p* = 0.445). Notably, the increase was significantly more pronounced in the LC_10_ than in the LC_25_, indicating a non-linear, dose-dependent response to the insecticide. The relative abundance of *Firmicutes* increased from 24.09% (CK3d) to 24.24% (LC_10_) and 23.30% (LC_25_) after 3 d of fipronil treatment. *Proteobacteria* showed significant increases at both time points (1 h: 34.88 and 26.20%; 3 d: 19.72 and 20.26%; *p* < 0.01) compared to controls (CK1h 12.21%, CK3d 12.14%). Conversely, *Bacteroidota, Actinobacteria* and *Verrucomicrobia* abundances were significantly reduced following exposure (1 h: 22.43, 21.04 and 1.19%, 10.23%; 3 d: 20.76, 20.66 and 11.75%, 12.71%; *p* < 0.01) relative to controls (23.44 and 17.37%, Figure 5A).

Genus-level analysis revealed treatment-dependent shifts in dominant taxa, with alterations in community structure occurring 1 h after exposure. (Figure 5B). While *Akkermansia* remained most abundant in controls (1 h: 13.33%; 3 d: 14.98%), fipronil exposure prompted *Lactobacillus* dominance after 1 h (LC_10_: 10.02%, LC_25_: 7.52%). Both *Lactobacillus* and *Escherichia* showed significantly elevated abundances in 1 h treatments (CK1h: 4.84, 1.37%, *p* < 0.001). *Streptomyces* was lower than that of the control group (5.01%). After 3 d exposure, *Akkermansia* dominance persisted, while the relative abundance of Lactobacillus was significantly higher than in controls (4.42%, *p* < 0.01). Concentration-dependent decreases were observed for *Akkermansia* (11.17 to 11.43%), *Muribaculum* (6.12 to 5.85%). The opposite was true for *Bacteroides* (3.98 to 4.05%) and *Streptomyces* (2.96 to 3.34%, *p* < 0.01).

### 3.6 Functional prediction analysis of microbial communities via PICRUSt 2

**Figure 6:**
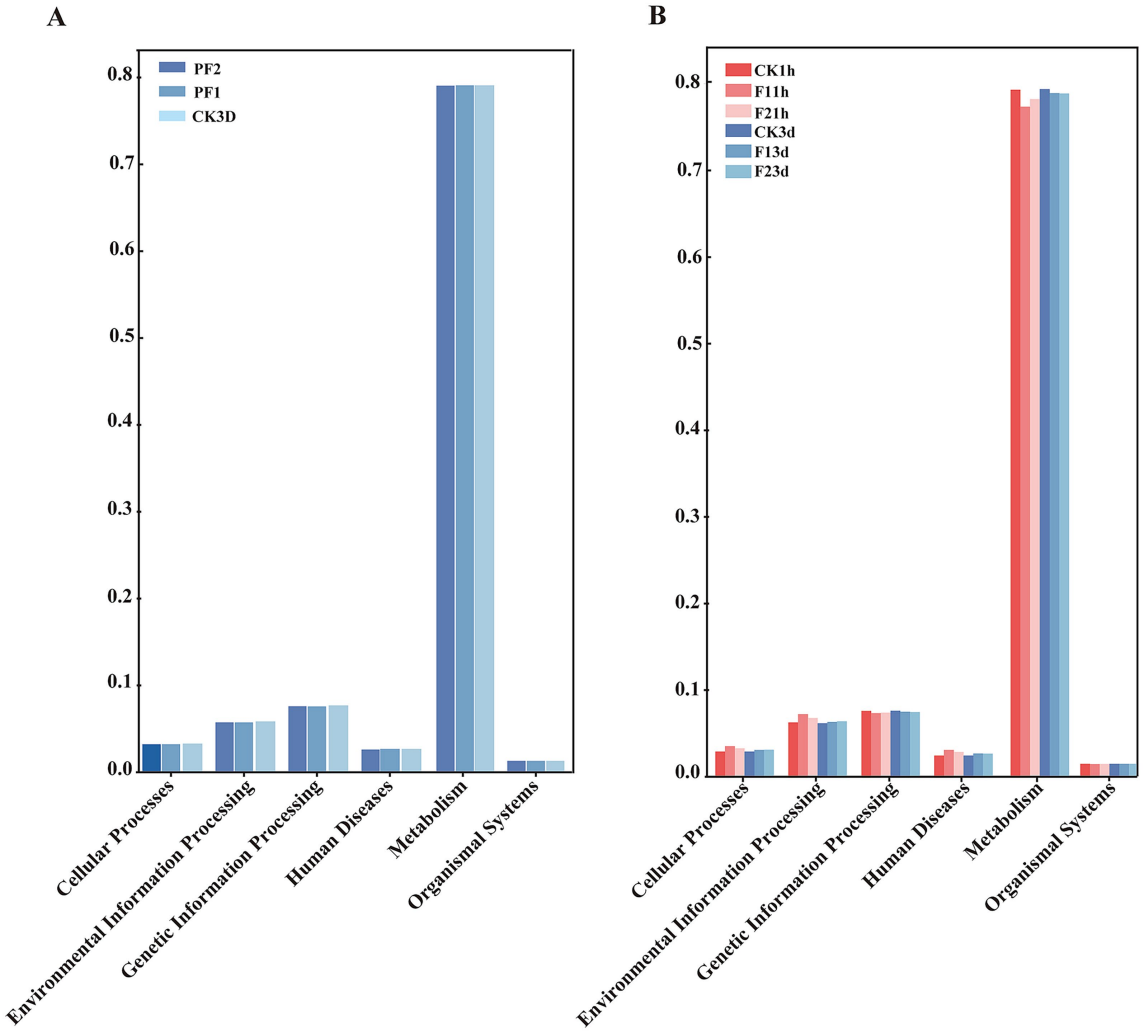
Functional analysis prediction. According to PICRUSt 2 function prediction, symbiotic bacteria in *B. communis* are primarily enriched in the top 6 biological KEGG functions. **(A)** *B. communis* larval. **(B)** Adult *B. communis.*

Functional prediction of the 16S amplicon sequencing data derived from *B. communis* associated microbiota was conducted using PICRUSt2 with reference to the KEGG database. The analysis revealed that the predicted functional genes were predominantly enriched in six major categories: cellular processes, environmental information processing, genetic information processing, human diseases, metabolism, and organismal systems. Notably, metabolic functions constituted the most substantial proportion (75%) of the predicted functional repertoire, suggesting that the microbiota associated with *B. communis* may play a critical role in its metabolism (Figure 6).

## 4 Discussion

As a broad-spectrum insecticide with multiple modes of action, prolonged residual activity, and versatile application methods, fipronil has become a cornerstone in modern agricultural pest management (Singh et al., 2021; Chen et al., 2022; Jaiswal et al., 2023). However, its ecological footprint extends beyond target pests, potentially affecting non-target organisms including beneficial insects such as predatory and parasitoid natural enemies through both direct and indirect exposure pathways (Stark et al., 2007; Biondi et al., 2012). Understanding these non-target effects is critical for optimizing integrated pest management (IPM) strategies that balance chemical and biological control (Tingle et al., 2003; Furlan et al., 2021; Lira et al., 2024). However, despite its ecological relevance, how sublethal fipronil exposure affects the symbiotic microbiome of parasitoid wasps like *B. communis* remains largely unknown. Our integrated approach, combining biological assays with 16S rRNA sequencing, provides the first evidence of fipronil-induced alterations in the microbiome of *B. communis*, highlighting a previously overlooked dimension of pesticide impact on parasitoid wasps. These findings underscore microbes can be used as an important reference for future evaluation of the safety of pesticides on insects that are not natural enemies of the target.

Our findings demonstrate that sublethal fipronil exposure (LC_10_ and LC_25_) induces significant developmental delays in *B. communis* larvae, with dose-dependent effects becoming particularly evident. We hypothesise that the observed developmental delays are likely the result of an energy trade-off. Larvae exposed to fipronil may divert energy resources originally allocated to growth and development toward detoxification processes and cellular repair, thereby mitigating the insecticide’s neurotoxic effects. This observation aligns with numerous reports documenting similar sublethal impacts of fipronil on non-target insect species (Desneux et al., 2004; Tosi et al., 2022; Du et al., 2024), reinforcing the broader ecological implications of pesticide use. The dose–response relationship observed in larval development with LC_25_ treatments showing greater prolongation than LC_10_ follows established patterns of insecticide-induced developmental delays (Sirota and Grafius, 1994; Kopit et al., 2021), suggesting conserved physiological responses across insect taxa. Notably, these developmental effects appear limited to the F0 generation, as we detected no significant impacts on larval duration, pupal development, or total lifespan in the F1 generation (Figure 1). This temporal limitation contrasts with known intergenerational effects of insecticides on other biological parameters such as parasitism efficiency and survival traits. For instance, bumblebees exhibited reduced lifespan following 48 h exposure to imidacloprid, thiamethoxam, and fipronil, whereas honeybees demonstrated increased survival rates after 4 h of exposure to sublethal doses of neonicotinoid insecticides (Lu et al., 2020; Blanc et al., 2020), highlighting the complex and parameter-specific nature of pesticide-induced transgenerational effects in insects.

The symbiotic microbiota of *B. communis* has undergone dynamic changes across developmental stages, reflecting distinct physiological demands. During the larval phase, Proteobacteria dominates the microbial community, likely facilitating nutrient acquisition through organic matter decomposition and metabolic conversion critical processes supporting the parasitic larval lifestyle (Dillon and Charnley, 2002). This microbial profile shifts markedly in adults, with Firmicutes emerging as the predominant phylum, consistent with its established role in carbohydrate metabolism and environmental adaptation (Meister et al., 2009; Wang et al., 2021). This distribution of dominant taxa aligns with the dominant phyla observed in the gut microbiome of honeybees (Wang et al., 2020; Zarrillo et al., 2025). Such stage-specific microbial transitions align with broader patterns observed in insect-microbe symbioses (Dillon and Dillon, 2004; Engel and Moran, 2013), suggesting an evolutionary conserved strategy for meeting developmental requirements.

The symbiotic microbiome of *B. communis* shows stage-specific modulation of microbial communities. While larval stages displayed increased microbial diversity coupled with decreased species richness, adults demonstrated concurrent increases in both diversity and richness indices. This differential response likely reflects the distinct physiological requirements and ecological roles of these developmental stages (Gao et al., 2021). The observed enhancement of microbial diversity, particularly in adults, may confer improved disease resistance and environmental resilience (Sheng et al., 2012), suggesting potential compensatory mechanisms in response to pesticide stress. These microbial shifts likely influence critical host functions including nutrient metabolism (Eichler and Schaub, 2002), immune regulation (Kikuchi et al., 2011), and physiological homeostasis (Meister et al., 2009). As predicted by our functional model, fipronil exerts an effect on the metabolism of parasitoids. The stage-specific nature of these microbial changes may underlie the differential developmental responses observed between larvae and adults, highlighting the intricate interplay between host physiology and symbiotic microbiota in mediating pesticide tolerance.

The core microbiome of *B. communis*, comprising *Vicinamibacter*, *Brevitalea, Akkermansia*, and *Muribaculum*, demonstrates sensitivity to fipronil exposure. Notably, Soil-acidophilic bacteria such as *Vicinamibacter* and *Brevitalea*, typically associated with acidic soils, may be acquired through the soil–plant-aphid trophic cascade (Pineda et al., 2010), highlighting the ecological connectivity of agricultural systems. This transfer exemplifies how edaphic microbial signatures can propagate across trophic levels, potentially influencing insect microbiomes. Of particular interest is the transient dominance shift from *Akkermansia* to *Muribaculum* in adults following 1 h LC_10_ exposure, potentially reflecting rapid microbial community restructuring in response to pesticide stress. Both genera play vital roles in gut barrier function and immune regulation (Macchione et al., 2019; Lei et al., 2023), suggesting that such perturbations could have cascading effects on host physiology. While prolonged (3 d) exposure did not alter the identity of the dominant genera, significant changes in their relative abundances were observed, indicating that fipronil primarily modulates microbial communities through quantitative rather than qualitative shifts. This finding supports the hypothesis that sublethal pesticide concentrations may influence host fitness by disrupting the delicate balance of symbiotic relationships rather than eliminating key microbial partners. The observed microbial dynamics could potentially impact critical host functions including nutrient assimilation, metabolic regulation, and immune competence, underscoring the need to consider microbiome-mediated pathways when evaluating pesticide effects on beneficial insects. These results contribute to a growing understanding of how agrochemicals may indirectly affect insect populations through subtle but ecologically significant alterations of their symbiotic microbiota.

## 5 Conclusion

Sublethal fipronil exposure (LC_10_ and LC_25_) induces stage-specific and concentration-dependent alterations in the endosymbiotic bacterial communities of *B. communis*, as revealed by 16S rRNA sequencing. Although larval development was prolonged in the F0 generation, no transgenerational effects on pupal duration or total lifespan were observed. The pesticide exposure dynamically modified microbial composition across developmental stages, affecting both relative abundance and diversity indices. However, these microbiome perturbations remained below the threshold for severe physiological disruption, suggesting resilience in the host-microbe symbiosis. Our findings demonstrate that sublethal pesticide exposure can cause subtle but ecologically significant microbial shifts, warranting further investigation into potential cumulative effects of prolonged or multigenerational exposure on host fitness.

## Data Availability

The data presented in the study are deposited in the NCBI repository, accession number PRJNA1356656.
