# Supplementary material for: Cross-generational ripples: sublethal fipronil exposure alters Binodoxys communis microbiome without lethal consequences
Source: Front Microbiol. 2025 Nov 21;16:1637234. doi: 10.3389/fmicb.2025.1637234 (PMC12679940; doi:10.3389/fmicb.2025.1637234)

**Supporting Information:**

**Table S1 16S rRNA Sequencing results**

**Table S2 Toxicity of fipronil to *Binodoxys communis***

**Figure S1 Dilution curve of the measured samples**

**Table S1 16S rRNA Sequencing results**

| \| **Sample ID** \| **Raw Reads** \| **Clean Reads** \| **Denoised Reads** \| **Merged Reads** \| **Non-chimeric Reads** \| \| --- \| --- \| --- \| --- \| --- \| --- \| |
| --- | --- | --- | --- | --- | --- | --- |
| \| **F11h-1** \| **79739** \| **79530** \| **75960** \| **70594** \| **65796** \| \| --- \| --- \| --- \| --- \| --- \| --- \| \| **F11h-2** \| **80007** \| **79804** \| **75760** \| **69329** \| **64087** \| \| **F11h-3** \| **80109** \| **79910** \| **76116** \| **70095** \| **65322** \| \| **F13d-1** \| **80025** \| **79826** \| **70236** \| **50669** \| **41236** \| \| **F13d-2** \| **79680** \| **79476** \| **70208** \| **50987** \| **42111** \| \| **F13d-3** \| **79979** \| **79793** \| **69009** \| **47321** \| **38090** \| \| **F21h-1** \| **80061** \| **79866** \| **72720** \| **57409** \| **49615** \| \| **F21h-2** \| **79757** \| **79568** \| **71485** \| **54859** \| **47163** \| \| **F21h-3** \| **79870** \| **79700** \| **71763** \| **56202** \| **48478** \| \| **F23d-1** \| **79921** \| **79739** \| **70162** \| **50012** \| **40714** \| \| **F23d-2** \| **80094** \| **79909** \| **70519** \| **50779** \| **41710** \| \| **F23d-3** \| **79898** \| **79673** \| **70099** \| **49939** \| **40588** \| \| **PF1-1** \| **79859** \| **79597** \| **70960** \| **56738** \| **46461** \| \| **PF1-2** \| **80128** \| **79845** \| **72008** \| **58369** \| **48541** \| \| **PF1-3** \| **80045** \| **79758** \| **71804** \| **58075** \| **48376** \| \| **PF2-1** \| **80255** \| **80046** \| **72556** \| **59503** \| **49652** \| \| **PF2-2** \| **79497** \| **79247** \| **71256** \| **57782** \| **47885** \| \| **PF2-3** \| **80010** \| **79743** \| **71768** \| **58153** \| **48000** \| \| **CK1h-1** \| **79858** \| **79666** \| **71756** \| **52350** \| **39667** \| \| **CK1h-2** \| **80168** \| **79998** \| **71610** \| **51824** \| **39303** \| \| **CK1h-3** \| **79738** \| **79560** \| **71581** \| **50941** \| **39171** \| \| **CK3d-1** \| **80002** \| **79834** \| **71067** \| **50897** \| **37359** \| \| **CK3d-2** \| **79939** \| **79752** \| **71683** \| **51913** \| **39275** \| \| **CK3d-3** \| **80281** \| **80099** \| **71861** \| **51756** \| **39180** \| \| **CK3D-1** \| **80004** \| **79734** \| **71936** \| **58485** \| **48644** \| \| **CK3D-2** \| **79840** \| **79602** \| **71117** \| **57272** \| **47717** \| \| **CK3D-3** \| **79941** \| **79680** \| **71653** \| **57816** \| **47875** \| |

**Table S2** **Toxicity of fipronil to *Binodoxys communis***

| **Insecticides** | **n** | **Slope ± SE** | **LC_10_（mg /L）**  **(95 % CL)** | **LC_25_（mg /L）**  **(95 % CL)** | **R^2^** | ***P*** |
| --- | --- | --- | --- | --- | --- | --- |
| **fipronil** | **360** | **2.32 ± 0.21** | **0.34**  **（0.16 - 0.54）** | **0.64**  **（0.37 - 0.91）** | **0.96** | **0.001** |

Note: LC_10_: 10% lethal concentration; LC_25_: 25% lethal concentration; 95 % CL: 95% confidence limits; R^2^: correlation coefficient; *P*: test of dispersion.

**Figure S1 Dilution curve of the measured samples**


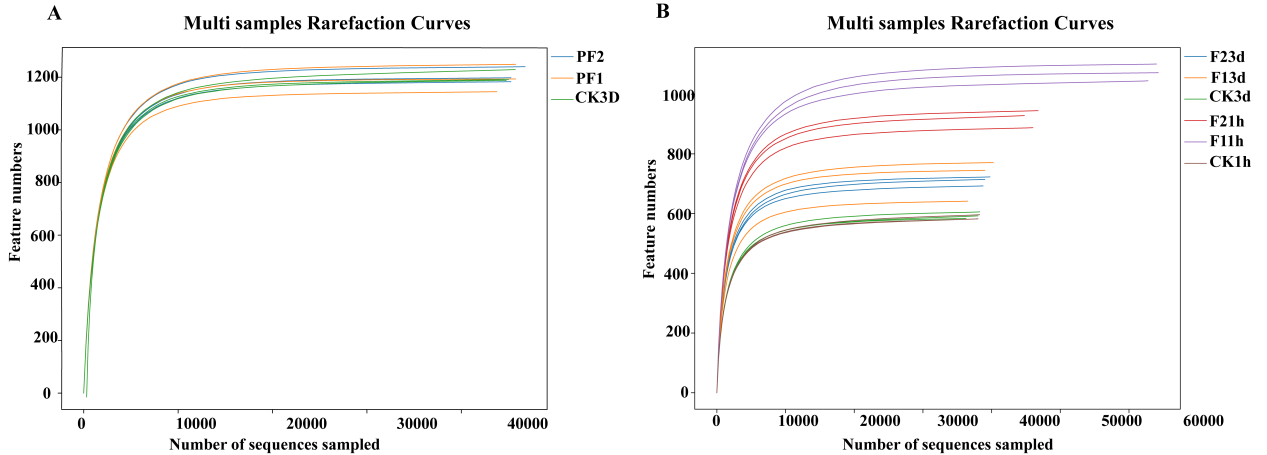

Supplement: Supplementary file 1 [file Table_1.DOCX]
